# Human papillomavirus expression in relation to biological behavior, Ki-67 proliferative marker, and P53 prognostic marker in Schneiderian papilloma

**DOI:** 10.25122/jml-2022-0312

**Published:** 2023-07

**Authors:** Shaymaa Fadhl Mohsin, Ban Al-Drobie

**Affiliations:** 1 Department of Oral and Maxillofacial Pathology, College of Dentistry, University of Baghdad, Baghdad, Iraq

**Keywords:** sinonasal papilloma, dysplasia, HPV, p53, Ki-67, IP: Inverted Papilloma, PCR: Polymerase Chain Reaction, mAbs: Monoclonal Antibodies, HPV: Human Papillomavirus, ISP: Inverted Sinonasal Papilloma, ESP: Exophytic Sinonasal Papilloma, ATP: Adenosine Triphosphate

## Abstract

Various malignant and benign tumors can arise in the sinonasal cavity, including inverted papilloma (IP), a benign neoplasm with unique clinical characteristics. However, the mechanisms involved in the recurrence, occurrence, and malignant transformation of IP remain debatable. This study aimed to investigate the impact of human papillomavirus (HPV) infections on IP by comparing the number of infections in cases with epithelial tissue dysplasia and explore the predictive role of proliferative and prognostic markers in dysplasia. Tissue blocks from 35 cases of sinonasal papilloma, collected between 2015 and 2021 from the laboratory archives of the Medical City of Ghazi Al-Hererri Hospital in Baghdad, Iraq, were immunohistochemically stained with monoclonal antibodies (mAbs) to detect Ki-67 and p53. A quantitative immunohistochemical analysis was conducted to analyze the results. Polymerase chain reaction (PCR) was performed to detect HPV genotypes 16/18 and 6/11 in the tissues. There was an insignificant increase in Ki-67 and p53 expression in inverted papillomas with dysplasia. HPV11 was the most prevalent genotype in 34.3% of the patients, followed by HPV16 and HPV18 in 31.4% of the patients for each virus. The least common virus detected was human papillomavirus 6 (8.6%), which did not show any significant association with the degree of dysplasia. Viral detection proliferation and apoptosis had no impact on tumor dysplasia amongst all the patients, showing no relationship with the evaluated cases.

## INTRODUCTION

Sinonasal papillomas, or Schneiderian papillomas, are benign epithelial neoplasms originating from the sinonasal (Schneiderian) mucosa. This mucosa is derived from the ectoderm and consists of basal cells, respiratory-type pseudostratified ciliated columnar epithelial cells, and mucus-producing goblet cells [1, 2]. Sinonasal papillomas account for 0.5%-4% of all nasal tumors, with an estimated annual incidence of 0.74-2.3 per 100,000 individuals [3-5]. Although these papillomas can occur across a wide age range, they are more common among older patients and typically have a predilection towards males. In general, sinonasal papillomas have a favorable prognosis; however, these can frequently recur, become locally destructive, and in some cases, undergo malignant transformation, often linked with metachronous or synchronous sinonasal carcinoma. The etiology of inverted sinonasal papilloma (ISP) is not yet fully understood, but human papillomavirus (HPV) has been detected in some cases [6]. HPV infections in ISP have been linked to an increased risk of progression to sinonasal squamous carcinoma [7]. Various parameters of cell kinetics have been studied to predict the disease course and clinical outcomes. An underlying characteristic of nasal IP and associated diseases is the loss of balance between programmed cell death and cell proliferation. Thus, an analysis of these two parameters is crucial for assessing the malignancy of these diseases. HPV infections play a critical role in the tumorigenesis of sinonasal inverted papilloma (IP). Integration of HPV into the host DNA triggers tumorigenesis by activating oncoproteins E7 and E6. The binding of E6 to p53 occurs via adenosine triphosphate, thereby leading to inactivation and degradation of p53. E7 binds and inactivates pRb, leading to the induction of p16, which plays a key part in the regulation of the transition of cells from the G1–S phase [8]. A detailed understanding of tumor biology is crucial for accurate oncological diagnoses. Modern immunohistochemical techniques allow the identification of proteins whose expression intensities change during carcinogenesis. The analysis of nuclear Ki-67 antigen expression (a marker of cell proliferative activity) and pro-apoptotic p53 protein can help assess the proliferation kinetics of tumor cells [9]. Therefore, the aim of this study was to investigate the relationship between low- and high-risk HPV infections and the increase in dysplastic epithelial tissues of sinonasal papilloma using the proliferative marker Ki-67 and prognostic marker p53.

## MATERIAL AND METHODS

### Tissue sample collection

35 formalin-fixed, paraffin-embedded tissue blocks were obtained from the archives of Ghazi Al-Hariri Hospital-Medical City between 2015 and 2022. The tissue blocks included samples from 9 females and 26 males. Relevant clinicopathological information, including gender, age, and location, was extracted from the accompanying histopathology reports. .

### Histopathological and immunohistochemical analysis

To confirm the diagnosis for each of the 35 patients, two expert pathologists reviewed the hematoxylin and eosin (H&E) stained sections. The tissue specimens were sliced into 4 mm thickness using a microtome, ensuring that the water bath was clean to avoid cross-contamination of tissues. The water bath surface was skimmed between cutting each block. Immunohistochemical (IHC) staining was performed following the deparaffinization of the tissue sections. The slides were incubated at 60°C for 1 hour using the Envision visualization system (K8000, Dako) with monoclonal antibodies targeting p53 (IS616, Dako, ready-to-use) and Ki-67 (IS626 Anti-Ki-67, Dako, ready-to-use). Heat-induced epitope retrieval was carried out using PT-LINK at 97°C. The Dako Autostainer Plus was used for immunohistochemical staining. Visualization was achieved using the Polymer Conjugate EnVision FLEX and DAB chromogen (K8000, Dako, Glostrup, Denmark). Counterstaining of the sections was performed using eosin and hematoxylin. Negative and positive controls were included for each immunohistochemical run.

### DNA extraction and PCR

The samples were deparaffinized using the Xylol-Ethanol method for DNA extraction and PCR. Genotyping was performed using virus-specific primers for various subtypes (Table 1). The PCR reaction mixture for HPV types 6, 16, 11, and 18 contained the PCR master mix component (Table 2) from the standard Accupower PCR premix kit. This kit included all the other required reagents to initiate the PCR reaction, including dNTPs, Taq DNA polymerase, MgCl2, Tris-HCl pH: 9;0, KCl, loading dye, and stabilizer. The PCR tubes were transferred to an Exispin vortex and centrifuged at 3000 rpm for 3 minutes. Subsequently, they were placed in a PCR thermocycler (Table 3). The PCR products were visualized on a 1% agarose gel, and positive results for specific HPV types were confirmed by the presence of corresponding fragments amplified with specific primers.

**Table 1:** Sequence of genotype-specific primers used in PCR

Primer	Sequence	Genotype
HPV 6F	TAG TGG GCC TAT GGC TCGTC	6 (280 bp)
HPV6R	TCC ATT AGC CTC CAC GGG TG	
HPV 11 F	GGA ATA CAT GCG CCA TGT GG	11 (360bp)
HPV 11 R	CGA GCA GAC GTC CGT CCT CG	
HPV16-E6F	CAG GAC CCA CAG GAG CGA CC	16 (380bp)
HPV16-E6R	ATC GAC CGG TCC ACC GAC CC	
HPV1S-E6F	GCT TTG AGG ATC CAA CAC GG	18 (440bp)
HPV1S-E6R	TGC AGC ACG AAT GGC ACT GG	

**Table 2:** Components of master mix for detection of ceramidase gene

Total	Material	Volume (µl)
1	Master Mix	12.5
2	Forward	1
3	Reverse	1
6	Template DNA	4
5	DH2O	6.5
Total		25 µl

**Table 3:** PCR program for detection of human papillomavirus (endpoint PCR for each primer)

No.	Stage	Cycle	Step	Temp.	Time
1	Initial Denaturation	1	1	92 °C	2 min.
2	Denaturation	45	1	92 °C	30 sec.
3	Annealing	*	2	45 °C	30 sec.
4	Extension	45	3	72 °C	20 sec.
5	Final Extension	1	1	72 °C	5 min.
6	Hold Phase			10 °C	

* : 56 for HPV 6; 58 for HPV 11; 55 for HPV 16-E6 and 60 for HPV 18-E6

### Statistical analysis

Descriptive statistics were used to analyze sociodemographic characteristics, including means, standard deviations, minimum and maximum values for continuous data, and numbers/percentages for countable data. Pearson correlation was employed to assess the correlation between continuous variables, while paired sample t-tests were used to compare the same population variable over time.

## RESULTS

The study sample consisted of 35 confirmed surgically excised paraffin-embedded tissue blocks of patients with sinonasal papilloma. The mean age of patients was 48±18.3 years, with males accounting for most cases (74.3%). The nose was the most common site of lesion presentation among the participants (85%).

**Figure 1:**
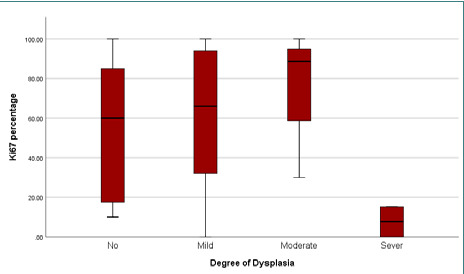
Mean Ki-67 expression across different degrees of epithelial dysplasia

**Figure 2:**
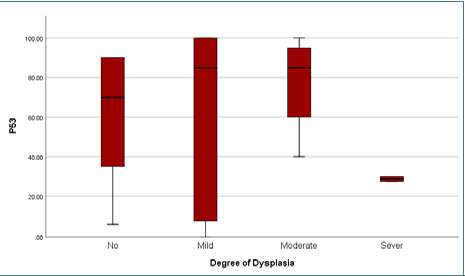
Mean P53 expression across different degrees of epithelial dysplasia

**Figure 3:**
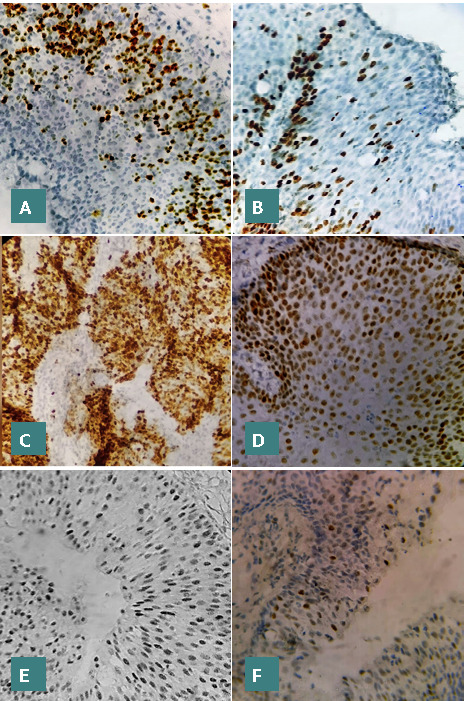
Positive immune expression with brown color (magnification 400X). (A) Ki67 in mild dysplasia, (B) ki67 in moderate dysplasia, (c) Ki67 in severe dysplasia, (d) P53 in mild dysplasia, (E) p53 in moderate dysplasia, (F) p53 in severe dysplasia

**Figure 4:**
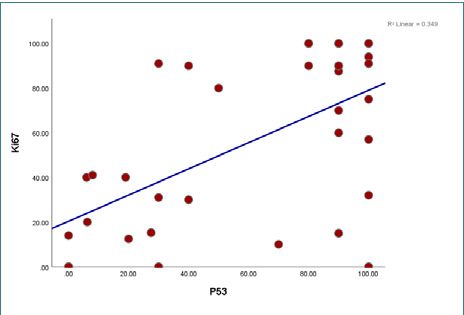
Correlation between Ki67 and P53

The findings revealed elevated expressions of Ki-67 and p53 markers, with mean expression levels of 56.1±37 and 61±37.7, respectively. The presence of HPV genotypes 6, 11, 16, and 18 were detected, but there was no statistically significant relationship with dysplasia (p-value>0.05) (Tables 4-7; Figures 1-3 A-F). However, a significantly moderate positive correlation was observed between p53 and Ki-67 (Figure 4). HPV was detected in 26 (74.3%) patients, indicating the presence of one or two HPV genotypes (6, 11, 16, 18). Additionally, 9 patients (25.7%) tested negative for HPV.

**Table 4:** Association between the degree of epithelial dysplasia and HPV 6 infection

			Degree of Dysplasia				Total
			No	Mild	Moderate	Sever	
**Hpv6**	**Negative**	Count	9	16	4	1	30
		%	30.0%	53.3%	13.3%	3.3%	100.0%
	**Positive**	Count	2	2	0	1	5
		%	40.0%	40.0%	0.0%	20.0%	100.0%
**Total**		Count	11	18	4	2	35
		%	31.4%	51.4%	11.4%	5.7%	100.0%

Chi-Square=4.9, p=0.35

**Table 5:** Association between the degree of epithelial dysplasia and HPV 11 infection

			Degree of Dysplasia				Total
			No	Mild	Moderate	Sever	
**Hpv6**	**Negative**	Count	8	12	2	1	23
		%	34.8%	52.2%	8.7%	4.3%	100.0%
	**Positive**	Count	3	6	2	1	12
		%	25.0%	50.0%	16.7%	8.3%	100.0%
**Total**		Count	11	18	4	2	35
		%	31.4%	51.4%	11.4%	5.7%	100.0%

Chi-Square=0.9, p=0.83

**Table 6:** Association between the degree of epithelial dysplasia and HPV 16 infection

			Degree of Dysplasia				Total
			No	Mild	Moderate	Sever	
**Hpv6**	**Negative**	Count	7	12	4	1	24
		%	29.2%	50.0%	16.7%	4.2%	100.0%
	**Positive**	Count	4	6	0	1	11
		%	36.4%	54.5%	0.0%	9.1%	100.0%
**Total**		Count	11	18	4	2	35
		%	31.4%	51.4%	11.4%	5.7%	100.0%

Chi-Square=2.3, p=0.51

**Table 7:** Association between the degree of epithelial dysplasia and HPV 18 infection

			Degree of Dysplasia				Total
			No	Mild	Moderate	Sever	
**Hpv6**	**Negative**	Count	8	14	1	1	24
		%	33.3%	58.3%	4.2%	4.2%	100.0%
	**Positive**	Count	3	4	3	1	11
		%	27.3%	36.4%	27.3%	9.1%	100.0%
**Total**		Count	11	18	4	2	35
		%	31.4%	51.4%	11.4%	5.7%	100.0%

Chi-Square=4.6, p=0.22

l group and vehicle group compared to the sham group (p<0.05) (Figure (5). Treatment with Iberin resulted in a significant reduction in IL-6 levels, approaching levels observed in the sham group (p<0.05).

## DISCUSSION

Schneiderian papillomas are relatively rare tumors that can occur within the nasal cavity, classified into three histological types: sinonasal inverted papillomas (SNIPs), oncocytic sinonasal papillomas (OSP), and exophytic sinonasal papilloma [10]. Typically, the malignant transformation of a sinonasal papilloma occurs via a dysplasia-to-carcinoma progression. Thus, dysplasia is a key histopathological finding in sinonasal papillomas and should be mentioned in surgical pathology reports. While dysplasia has been observed in ISP and OSP, it is not well described in ESP [11].

The progression of an ISP to dysplasia and malignancy may be due to a secondary HPV 16/18 infection, loss of tumor suppressor gene, integration of HPV 16/18 and HPV 6/11, or a combination of these aspects [12]. In our study, we found no significant relationship between dysplasia and HPV infections, which is consistent with previous findings [13]. Other studies have reported different rates of HPV infections in SNIP with and without dysplasia but without identifying any statistically significant relationship. Some studies were unable to detect HPV DNA in dysplasia and carcinoma associated with IP specimens, while others have shown varying HPV detection rates and contradictory results regarding the role of HPV infections in malignant and recurrent transformation [14-16]. Overall, the inconsistency in the results of previous studies may be attributed to factors such as DNA degradation in paraffin-embedded tissues, leading to false negative results, as well as geographical and racial differences.

The proliferation antigen Ki-67 is employed to evaluate the proliferation index of a cell population and for grading dysplasia concerning cervical biopsies [17]. It has been speculated that Ki-67 can directly impact the cell cycle in the proliferation phase. It could also interfere with the p21, p53, and p27 tumor suppressor genes, enabling modulation of the cell cycle by interfering with the checkpoint of the G1 phase [18-20]. In the current study, increased proliferation of epithelial cells was seen in SNP with dysplasia and SNP, but it did not show any significant correlation, which agrees with earlier studies [21-22]. However, some researchers have reported an increase in the expression rate of Ki-67 with an increase in dysplasia grading [23]. These discrepancies may be attributed to differences in the methods used to evaluate Ki-67 expression or the criteria employed for counting positive tumor cells [24, 25].

Detecting p53 in normal tissues is challenging due to its very short half-life. However, in response to mutation, the modified p53 protein expression increases, and it can also be easily detected via IHC [24]. In our study, we observed a high percentage of p53 expression in non-dysplastic benign IPs and dysplastic tissues, with increased expression associated with higher grades of dysplasia. Nevertheless, there was no significant difference between the degree of dysplasia and the mutated genes, possibly due to the limited number of patients involved in the study. This finding was in line with other studies [25, 26]. In contrast, another study [23] demonstrated significantly increased p53 staining in SNIP with severe dysplasia, invasive carcinoma, and SNIP with carcinoma compared to control nasal mucosa. They suggested that p53 testing could be used for screening papilloma lesions and detecting carcinoma or dysplasia. Furthermore, a positive correlation was observed between p53 expression and the Ki-67 index in cases of SNIP with dysplasia and SNIP, in line with previous studies [27]. The detection of HPVs in 74.3% of cases, along with the high expression rates of Ki67 and p53 (100%), are notable strengths of our study. However, it is important to acknowledge the limitation of the small sample size due to the rarity of the tumor and the withdrawal of several cases from the archives of the medical center. As a result, the correlation between these variables and dysplastic changes in the epithelial tissue was insignificant.

## CONCLUSION

The expression of Ki-67 and p53 proteins, as well as the presence of viral genomes, did not have a significant impact on the biological behavior of sinonasal papillomas with dysplasia. These factors do not serve as prominent indicators of tumor aggressiveness and do not contribute to a reliable prognostic assessment of SNP.
